# Sleep Complaints in the Psychiatric Hospital: A Qualitative Study of Nurses and Psychiatrists’ Approaches to Sleep Management in a Swiss Psychiatric Hospital

**DOI:** 10.3390/clockssleep8010005

**Published:** 2026-01-20

**Authors:** Maria Dalmau i Ribas, Geoffroy Solelhac, José Haba-Rubio, Julien Elowe, Véronique Griffith

**Affiliations:** 1 Hôpital Psychiatrique de Prangins, Department of Psychiatry, Centre Hospitalier Universitaire Vaudois (CHUV), 1011 Lausanne, Switzerland; julien.elowe@chuv.ch; 2 Centre D’Investigation et de Recherche Sur le Sommeil (CIRS), Centre Hospitalier Universitaire Vaudois (CHUV), 1011 Lausanne, Switzerland; geoffroy.solelhac@chuv.ch (G.S.); jose.haba-rubio@chuv.ch (J.H.-R.); 3 Faculty of Life Science & Medicine, King’s College London, London WC2R 2LS, UK; veronique.griffith@manchester.ac.uk

**Keywords:** sleep, insomnia, hypnotic, sedative, benzodiazepine, Z-drug, CBT-I

## Abstract

Insomnia symptoms are very common among psychiatric inpatients and can increase the risk of suicide in this population. However, little is known about how psychiatrists and nurses manage insomnia symptoms in psychiatric inpatients. This study aimed to investigate the views, opinions, and experiences of psychiatrists and nurses regarding inpatients’ sleep complaints in a Swiss psychiatric hospital. This qualitative study used individual semi-structured interviews with a purposive sample of psychiatrists and nurses working in a Swiss psychiatric hospital. Interviews were audio-recorded, transcribed verbatim, and analysed manually using inductive thematic analysis. Ten participants (six psychiatrists and four nurses) were interviewed. Three overarching themes were identified: identifying and classifying sleep complaints, the decision-making process, and the actions taken to respond to the complaint. Insomnia symptoms were approached by psychiatrists and nurses in a highly heterogeneous, non-evidence-based manner, with a lack of adaptation of CBT-I leading to overmedication. This heterogeneity may be explained by the diversity of underlying problems associated with insomnia symptoms, the lack of hospital-specific guidelines, and the fact that current guidelines focus mainly on chronic insomnia and do not fully account for the complexity of psychiatric inpatients.

## 1. Introduction

Insomnia symptoms are very common in the general population, with around 34% of the adult European population presenting with insomnia symptoms [1] and around 10% meeting the diagnostic criteria for insomnia disorder [2]. Untreated insomnia is associated with cardiovascular and metabolic comorbidities and negatively impacts health-related quality of life [3]. The prevalence of insomnia symptoms is considerably higher in people with mental disorders [4]. Among this population, one third suffer from a chronic insomnia disorder [5].

Insomnia disorder is defined as subjective difficulties falling asleep, staying asleep, or early awakening more than three times per week for at least three months, with complaints of impaired daytime functioning [6,7,8]. In contrast, insomnia symptoms designate clinical presentations that do not meet the diagnostic criteria for insomnia disorder because symptoms are acute or occur in the absence of daytime impairment [9].

Hospitalised patients may also present insomnia symptoms due to environmental and patient-related factors [10,11], and several structural and organisational modifications have been shown to improve patients’ sleep [12]. Specific guidelines on how to manage insomnia symptoms in hospitals do not exist at a European level, and insomnia guidelines recommending Cognitive Behavioural Therapy for Insomnia (CBT-I) as a first-line treatment are not usually applied in the hospital context [13,14], although promising interventions are being tested to implement an adapted form of CBT-I among psychiatric inpatients [15]. For this reason, sleep-inducing drugs are widely prescribed among inpatients [16], with approximately 40% of Swiss inpatients receiving at least one sleep-inducing drug during their hospitalisation [17]. The use of these drugs among patients with psychiatric comorbidities is much higher than in the general population [18], and about 90% of psychiatric inpatients report insomnia symptoms during hospitalisation, which has been associated with increased suicidal ideation [19].

Benzodiazepines and z-drugs are effective in treating acute insomnia [3]. Nevertheless, their effect size is generally small, while the risk of adverse effects is substantial [20]. Despite a risk–benefit balance that advises against their use [21], healthcare professionals tend to overestimate the benefits of these drugs while underestimating their associated risks [13], which include tolerance, rebound insomnia, and dependency [22].

Little is known about how insomnia symptoms are managed in psychiatric inpatients, although this population is at higher risk of insomnia disorder and sleep-inducing drug use [18]. Studies focusing on psychiatrists’ and nurses’ views, opinions, and experiences on this topic are lacking, even though they are the professionals who decide on prescribing or request prescriptions for sleep-inducing drugs. Some authors have highlighted the importance of conducting qualitative studies in this field to gain a broader understanding of the problem [13]. A better understanding of their approaches to inpatients’ insomnia symptoms could positively impact psychiatric inpatient care and promote the development of hospital-specific insomnia guidelines.

This study investigates the views, opinions, and experiences of nurses and psychiatrists working in a Swiss psychiatric hospital regarding their approach to patients’ sleep complaints. Participants were asked about “sleep complaints” rather than “insomnia symptoms” to assess whether sleep complaints other than insomnia symptoms (such as hypersomnia symptoms, respiratory symptoms, or circadian symptoms) were discussed, and to avoid framing responses in terms of diagnostic categories rather than patients’ subjective complaints [23].

## 2. Results

Ten participants were recruited for the study (six psychiatrists and four nurses) from a total of 100 people invited. The sample was heterogeneous and consisted of junior psychiatrists, chief psychiatrists, night nurses, and chief nurses. Relevant demographic data about the participants are summarised in Table 1.

Through thematic analysis, three overarching themes were identified: identifying and classifying sleep complaints, the process of decision-making, and the actions taken to respond to the complaint. Data saturation was reached, as the last three interviews did not add new information [24].

### 2.1. Identifying and Classifying Sleep Complaints

All participants highlighted the importance of identifying the problem underlying sleep complaints before considering solutions. They agreed that obtaining a complete sleep anamnesis and making sense of the context was indispensable. This included asking patients about their daytime and sleep routines to evaluate their sleep hygiene status. P2 mentioned: *“Once you understand what happens, is there something that the person can do to improve [their sleep]? Typically in the hospital, are they doing activities or are they just laying down or sitting the whole day? Do they get tired? At what time are they going to sleep? Do they eat, do they drink coffee? So already these questions about… purely about sleep hygiene”*.

Most participants noted that sleep complaints encompassed a heterogeneous group of problems that could be divided into three categories: acute sleep problems linked to the hospital environment, acute sleep problems linked to acute psychiatric manifestations, and chronic sleep problems present before hospitalisation. For P6, the environment alone could explain the appearance of sleep complaints: *“It is already stressful to be in the psychiatric hospital, I think. It is the most important factor [explaining poor sleep]. Of course, nurses stopping by every hour with their pocket lamps to see if everyone is sleeping, that is extraordinary!”*. Others mentioned that although nurses’ hourly visits were performed for security reasons, they disrupted inpatients’ sleep. P7, on the other hand, explained that sleep complaints were usually linked to the underlying psychiatric problem: *“[In a crisis], we consider that the sleep disorder is secondary to the psychiatric pathology. We are going to try, as fast as possible, to impact the cause, that’s to say, the psychiatric pathology”*. Nevertheless, it was also recognised that some patients presented with chronic sleep complaints unrelated to hospitalisation, as exemplified by P1: *“But on the other hand, there are patients who have chronic sleep disorders, and these patients, they need a baseline treatment for their sleep disorder.”*

### 2.2. The Process of Decision-Making

Participants identified two factors influencing the decision-making process: patient-related factors and factors related to psychiatrists and nurses.

Concerning patients, the main diagnosis was identified as one of the most important factors. P3 mentioned: *“It changes a lot if it is someone depressive or someone psychotic. […] Also if it is someone known for substance dependence in the past. Because we don’t want to create other dependencies.”* Other participants mentioned that most molecules used for sleep are potentially addictive, especially among patients with dependencies.

Participants also considered the severity of the condition, comorbidities, patients’ current and past medication, and the side-effect profile of sedative-hypnotic medications. For P7, the severity of the condition informed her practice: *“[We can use non-pharmacological approaches] among the mildest cases. So, clearly, not among hospital cases. […] With a patient who is extremely depressed, I will not take the risk of losing three or four days with phytotherapy, because it is not going to work, and I will go straight to a benzo[diazepine].” “Not wanting to take the risk”* was mentioned by many as a main reason for adopting a pharmacological strategy from the outset.

Patients’ expectations, attitudes, and demands, as well as their socio-cultural background, were also identified as important. P5 said: *“There are patients who are against drug strategies, who are much more in demand of behavioral strategies. And there, my resources would be to go more into behavioral strategies of CBT [cognitive behavioural therapy], with relaxation exercises, with breathing exercises”*. Nevertheless, most participants believed patients preferred fast-acting drugs. P7 illustrated this: *“One factor that I find quite annoying is the competition between patients. It’s the discussion of ‘and you, what do you have to sleep?’. And it doesn’t miss. ‘My roommate has this and I see he sleeps like a king’”*. Others, like P6, recognised that both realities coexisted: *“There is a part of people who are open to [listening to recommendations on sleep hygiene], but the other not at all. They just ask for the molecule, they just ask for a tablet. They don’t care that they might be using too much screen,* etc.” P9 suggested that socio-cultural background might explain these differences: *“It may not be very politically correct to say that, but I think that the socio-cultural level of the patients influences a little bit too. […] A patient who can question the treatments […] perhaps we will end up with a benzo[diazepine] less quickly […] If, on the other hand, it’s someone who says ‘I don’t understand anything about drugs, I’m not interested’, we’re going to tell ourselves, ‘we’re going to give him benzo[diazepine]s and at least he’s not going to bother us like that’. It’s not like that black and white, but there must be something on that level, yes”*.

Therapeutic alliance was also mentioned as influential. P9 said: “*What makes us act differently between one patient and another is unfortunately somehow the therapeutic alliance that we may or may not have established with them. […] With the ‘good patient’ we would be quite empathetic […] And the patient with whom we have more counter-attitudes […] we send them away.”*

Regarding professionals’ factors, communication and collaboration between nurses and psychiatrists were described as key. P8 illustrated this: *“[Managing sleep complaints] is something that I do with the on-call doctor […]. I am not alone during the night […] We are the eyes and ears of the doctor, and afterwards it is a discussion*”. Education and training were also identified as relevant. P3 stated: “*In psychiatry we do not have many instructions regarding sleep […] it would be good if we all had the same training and not do as you feel or in relation to your experience*”. Role modelling was also mentioned. P8 said: “*When [name of a chief doctor] left, we moved on to another trend […] we are more on melatonin, Redormin [phytotherapy], Circadin [slow-release melatonin]*”. A similar view was shared by P10.

No participant mentioned guidelines as influencing practice. P1 noted: “*I am not sure that having protocols all the time is helpful*”. P9 explained that recommendations were difficult to apply in hospital settings: “*In the hospital, we have to do a little bit the opposite of what we are told to do at home when we have sleep problems […] We are a little stuck with the advice that we give and the practice that prevents it”*.

### 2.3. Action Taken to Respond to the Complaint

All participants identified two different ways of responding to sleep complaints: pharmacological and non-pharmacological strategies. Everyone highlighted the importance of pharmacological treatments, and many considered them the most appropriate response in the hospital setting. P1 mentioned: “*Non-pharmacological approaches can also be used, but it may not be enough for patients who really have difficulty falling asleep […]. With people who have recalcitrant insomnia […], the attitude, the non-pharmacological approaches, they will be useless*”. Some participants claimed that non-pharmacological strategies were not appropriate in the psychiatric hospital, such as P2: “*When to use non-pharmacological strategies? Not in the hospital (laughs). In the outpatient clinic*”.

Medication was perceived to act faster and more effectively. The choice of molecule was highly influenced by the main diagnosis. Most participants reported using sedative antidepressants such as mirtazapine or trazodone for patients with sleep complaints and depressive symptoms, as illustrated by P1: “*We have patients with sad mood or a depressive disorder […], and when there are problems falling asleep or remaining asleep, the doctors like to introduce either Trittico [trazodone] or Remeron [mirtazapine], because we know that these are antidepressant treatments that have an effect on anxiety, on sleep, on appetite… So there are drugs like that have an effect on several problems of the patients*”. In contrast, participants reported using sedative antipsychotics when sleep disturbances occurred alongside psychotic episodes, as P7 explained: “*In the hospital, it is very frequent, because of the pathology from the spectrum of the psychosis, to use neuroleptics, what I call sedative [neuroleptics]. In the outpatient clinic, I have never used a neuroleptic as a first-line treatment as a hypnotic drug*”.

Participants were generally more inclined to use benzodiazepines for patients suffering from anxiety disorders and for those who were already receiving benzodiazepines for other indications, as illustrated by P6: “*Of course, if there is someone who is already very very familiar with benzo[diazepine]s, as you know, we can prescribe them. So here it is, if someone asks you ‘can you prescribe me a [hypnotic]?’ I do. I don’t think too much. So [it would be] someone who certainly already has a long list of medications. A patient who is well known to us. Someone who says ‘I have been taking a sleeping pill for years’. At that point, I say okay. I’m not going to try Redormin [phytotherapy]*”. Z-drugs were described as adjuvant treatments when the intervention addressing the psychiatric cause had failed or was insufficient, as P3 stated: “*It is true that it is rather the nurses who call us [during the night] to tell us ‘The patient has trouble sleeping. He is known for…’ and either he’s actually the type of patient for whom it is linked to the anxiety, and then I’m going with benzodiazepines, or he is someone who has several things already, and then it is rather the zolpidem, the Stilnox [slow-release zolpidem]… things like that*”.

Although most participants distinguished sleep complaints into three subgroups, only a few considered whether the sleep complaints was acute or chronic when prescribing medication. Some participants considered that even sleep complaints related to the hospital environment required treatment with a sedative-hypnotic, as illustrated by P3: “*I imagine that when the nurse calls me it’s either the patient who’s really ruminating in his room, or disturbing the other neighbour, or he’s just starting to walk down the hall and… Yes, it is the patient plus his environment. So I think it’s both things. To prevent the situation from getting worse, it’s true that it’s easier to say ‘Ah, well, we’re going to do that’ [give a hypnotic medication]*”. Other participants, such as P5, thought it was preferable to address environmental factors whenever possible before considering medication: *“I think that, from an environmental point of view, there is still the possibility of changing rooms. If the neighbour in the room is very noisy and very disturbing for the patient who reports sleep problems, and it is not possible to change the neighbour in the room, there are also individual rooms. […] Because telling the patient that his room must be dark at night… We know that sometimes the hospital environment does not have all the structures we would like*”.

Many participants considered it good practice to start with phytotherapy or melatonin and then escalate treatment if the problem persisted. As P3 explained: “*I start by giving corrective hygiene measures and seeing if that helps or not. And then, I start… Well, personally, I like to start with more natural things [phytotherapy], and after that melatonin, then really if it is something chronic or it is not going well, I try to go little by little. If it is not melatonin, then I try to see if it is something related to anxiety, and there, for example, I would start with Temesta [lorazepam]. If it is not that, if it is really someone coming for a disorder that is more chronic, I always try to leave the melatonin and then add something else, maybe stronger, like zolpidem, for example*”.

## 3. Discussion

In this study, we investigated the views, opinions, and experiences of nurses and psychiatrists working in a Swiss psychiatric hospital regarding their approach to patients’ sleep complaints. To the best of our knowledge, this is the first qualitative study on this subject.

First, we observed that although participants were asked about “sleep complaints” in general, all participants assumed they were being asked specifically about “insomnia symptoms.” This may be explained by the fact that insomnia symptoms are present in up to 90% of psychiatric inpatients [19] and are therefore likely the most frequent sleep complaint in psychiatric hospitals. Nonetheless, other sleep disorders, such as obstructive sleep apnea, nightmare disorder, restless legs syndrome, or circadian rhythm disorders, may also be common and remain undiagnosed in this population [25,26,27].

The main finding of this study is that sleep complaints in the psychiatric hospital encompass three different types of problems: acute insomnia symptoms arising from the hospital environment, acute insomnia symptoms secondary to the underlying psychiatric pathology, and chronic insomnia symptoms. This is an important finding, as the management of sleep complaints may differ for each category.

Despite this distinction, most participants did not use different strategies depending on the type of sleep complaints. Most believed sedative-hypnotics to be very effective, a perception also described in another study [13], which concluded that nurses and doctors tended to overestimate the effects of these drugs. Although that study was conducted in a general hospital, the present study found similar perceptions among psychiatrists and psychiatric nurses. The widespread use of sedative-hypnotics may be partly explained by the fact that European insomnia guidelines recommending CBT-I as a first-line treatment focus primarily on chronic insomnia and previously considered insomnia symptoms lasting less than three months as not requiring systematic treatment, as they may resolve spontaneously [28].

Nevertheless, sleep complaints in the psychiatric hospital are more complex, as acute insomnia is a well-known risk factor for suicide [19]. Most people who die by suicide suffer from psychiatric disorders and have been hospitalised in a psychiatric hospital at least once [29], suggesting that suicide prevention in hospital settings is particularly important [30]. This may explain why, despite knowing a wide range of non-pharmacological strategies, most participants reported that they would “not take the risk” and would be more inclined to use medication as a first-line treatment. However, this assumption may be incorrect, as some studies suggest that CBT-I not only improves insomnia symptoms but also symptoms of the comorbid mental disorder [31]. Moreover, a recent study by Schneider et al. [15] demonstrated the feasibility of implementing an adapted CBT-I in an inpatient psychiatric facility for a broad spectrum of psychiatric disorders, using existing hospital resources. The same study showed a trend towards reduced time in bed, increased subjective total sleep time, and improved sleep efficiency, suggesting a reduction in insomnia severity. On the other hand, sedative-hypnotics may produce faster treatment effects than CBT-I alone [32], and therefore individual risks and benefits must be considered when deciding whether to prescribe an adjuvant sedative-hypnotic.

Interestingly, despite CBT-I being a strong evidence-based first-line treatment for insomnia [2], none of the participants mentioned it as a treatment option for insomnia symptoms in the psychiatric hospital. A recent study found that most Swiss general practitioners have very limited knowledge of CBT-I and that very few know a CBT-I provider [33]. Although participants were not directly asked about CBT-I, the fact that none mentioned it may suggest that knowledge of CBT-I among participants was also very limited. Consistently, a recent study found that the use of CBT-I in psychiatric hospital was very low [14].

Nevertheless, some evidence-based components of CBT-I [34] were identified by participants as non-pharmacological strategies. Psychoeducation about sleep was not mentioned explicitly, although participants reported recommending sleep hygiene measures. However, sleep hygiene alone should not be proposed as a treatment for insomnia, as it does not constitute an evidence-based intervention and may even increase the perceived threat of not being able to sleep [2]. Relaxation therapy was mentioned by one participant and may indeed be effective in facilitating de-arousal and acting as a reconditioning agent [34]. Cognitive therapy was not mentioned by any participant. Sleep-restriction therapy (SRT), which involves restricting sleep opportunity to increase sleep pressure and consolidate sleep [35], was not mentioned by any participant. Stimulus control therapy (SCT), an operant conditioning strategy aimed at re-establishing the association between bed and sleep [36], was mentioned by only one participant, despite these two components being hypothesised as the most effective elements of CBT-I [37].

Conversely, most participants suggested insomnia treatments that are not evidence-based [2], such as phytotherapy, melatonin, and antipsychotics. Among the most commonly used medications, participants mentioned benzodiazepines, Z-drugs, and trazodone. Although these treatments are included in the most recent European guidelines [2], they are recommended only after CBT-I has been ineffective. Some participants highlighted the difficulty of discontinuing benzodiazepines and Z-drugs after hospitalisation and suggested that longer courses of treatment were often necessary.

Another important finding is that practices varied widely among participants, regardless of their experience in psychiatry. Many adopted strategies based on personal experience rather than scientific evidence. One possible reason is that insomnia guidelines are often based on evidence that does not reflect real-life patients, such as those with a single diagnosis or without medication [38], which contrasts with psychiatric inpatients who typically present with high levels of comorbidity and polypharmacy [39]. Another key reason may be that psychiatrists and psychiatric nurses are not specifically trained to treat insomnia symptoms or insomnia disorder, as several participants noted. Indeed, a lack of trained professionals has been identified as one of the main barriers to CBT-I delivery [40].

Finally, participants identified several hospital-related factors that negatively impacted inpatients’ sleep quality. Most of these factors were consistent with the existing literature. For example, Heinemann et al. [13] described sleeping in an unfamiliar environment, sharing rooms, and nighttime nursing interruptions as factors that impair sleep. Some factors, however, appear to be specific to psychiatric inpatients, such as hourly nursing checks performed for safety reasons. A recent study conducted in UK psychiatric facilities using actigraphy concluded that sleep was likely unnecessarily fragmented due to these checks and suggested that, although necessary for safety, they should be individualised to minimise their impact on sleep [25].

In terms of transferability, some findings may be applicable to other contexts [41]. For instance, the division of sleep complaints into three categories may be relevant to general hospitals, where structural factors affect sleep [13], patients experience acute IS due to their underlying condition [3], or suffer from chronic insomnia predating hospitalisation [15]. Similarly, reflections on tailoring sleep complaints management to patients’ diagnoses may also apply to psychiatric outpatients, who frequently experience IS in the context of multiple psychiatric comorbidities [31].

### 3.1. Limitations of the Study

The main limitation of this study concerns the primary researcher’s (MDR) positionality. She knew all participants prior to the study, which may have introduced bias [42]. Most participants were aware of her special interest in sleep medicine and may have known how she typically managed sleep complaints. Because of this positionality, participants may have assumed that the researcher had specific expertise in the subject. To mitigate this risk, a brief introduction was provided before each interview, emphasising that the focus was on participants’ views, opinions, and experiences. In addition, questions were framed to ask about “what is done” rather than “what the participant did,” which may reduce social desirability bias [43]. Perceiving the researcher as an insider may also have had benefits [44], such as participants feeling more comfortable discussing sensitive topics due to perceived empathy.

Another important limitation is that general nurses were not included in the sample, despite representing the largest group invited to participate. The reasons for this are unknown, but it is possible that they perceived sleep complaints management as outside their area of expertise or primarily the responsibility of night nurses. Although data saturation was reached, it remains unclear whether including this subgroup would have yielded additional insights [45].

Finally, the fact that coding and data analysis were conducted by a single researcher represents a further limitation, as reliability would likely have been enhanced if two or ideally three coders had been involved [46].

### 3.2. Future Perspectives

The present study offers an opportunity to identify pathways for improving the management of sleep complaints in psychiatric hospital settings. As several participants highlighted a lack of specific knowledge about sleep and insufficient training in sleep management, it appears essential to strengthen education on sleep and insomnia across professional training pathways. In particular, undergraduate and postgraduate curricula in medicine and nursing should more systematically integrate evidence-based approaches to sleep assessment and treatment.

Clinical psychologists, who are not part of the clinical staff in many psychiatric hospitals, could also play a key role in improving sleep care in inpatient psychiatric settings. Consequently, postgraduate psychology training programmes should include formal education in sleep medicine and cognitive behavioral therapy for insomnia (CBT-I). As recommended by the European CBT-I Academy, several specialized training courses are already available across Europe and could provide healthcare professionals who have not previously received such training with the necessary foundational competencies.

Beyond individual training initiatives, future efforts should also focus on developing interdisciplinary sleep management protocols within psychiatric hospitals. This includes implementing structured screening tools for sleep complaints and integrating CBT-I-based interventions into routine inpatient care, with the aim of improving sleep outcomes and overall patient well-being.

## 4. Materials and Methods

### 4.1. Study Design and Setting

This qualitative study used semi-structured interviews with nurses and psychiatrists working in a Swiss psychiatric hospital. All participants were recruited from a single centre, the Hôpital Psychiatrique de Prangins, a psychiatric hospital serving the western region of the Centre Hospitalier Universitaire Vaudois (CHUV; Lausanne University Hospital). The hospital has 92 inpatient beds distributed across five units (four adult units and one psychogeriatric unit) and serves a mostly rural population of approximately 200,000 inhabitants. At the time of the study, the institution employed 75 nurses and 25 psychiatrists.

The hospital provides acute psychiatric inpatient care for individuals with a range of primary diagnoses, including mood disorders, psychotic disorders, substance use disorders, and personality disorders presenting with suicidal crises. The average length of stay is approximately three weeks for adult hospitalisations and four weeks for psychogeriatric hospitalisations. The psychogeriatric unit is a mixed ward combining old-age psychiatry and dementology.

The four adult inpatient units each comprise 17–18 beds and admit acute patients. During daytime shifts, staffing typically includes approximately three nurses per unit, supported by two nursing assistants, while night shifts are covered by one nurse per unit and one nursing assistant shared between two units. Each pair of adult units is staffed medically by two resident doctors, one senior doctor, and one consultant psychiatrist. The psychogeriatric unit has 20 beds and a comparable medical staffing structure, with a higher number of nursing assistants depending on patient needs. At night, the psychogeriatric unit is staffed by one nurse and one nursing assistant. Across the hospital, medical on-call coverage consists of one junior doctor on site and one senior doctor providing supervision by telephone.

The Consolidated Criteria for Reporting Qualitative Research (COREQ; [47]) were considered in the design and reporting of the study to enhance methodological rigor and transparency [48].

### 4.2. Participants

#### 4.2.1. Recruitment

Chief nurses and psychiatrists were approached by the researcher (MDR), who asked them to distribute the invitation letters in their respective units. Potential participants were asked to contact MDR via email. Participants were recruited between May and June 2022, and interviews took place between June and July 2022.

#### 4.2.2. Selection

The study aimed to recruit a purposeful sample of 8–12 psychiatrists and nurses working in a specific psychiatric hospital in Switzerland, as data saturation had been reached with this number of participants in similar studies [49]. The sample was selected using a maximum variation sampling strategy, and therefore participants were selected considering their years of experience in psychiatry (to include both training and trained psychiatrists), their position, and their gender, to provide a sample as heterogeneous as possible so that different views could be captured [50]. Inclusion criteria included working in that hospital at the time of the study, having worked at least one night in that hospital, and speaking French fluently, the language used in the interviews.

### 4.3. Interviews

Semi-structured interviews were conducted by the researcher. They were audio-recorded and transcribed manually verbatim. Interviews lasted between 20 and 40 min. The participants’ views, opinions, and experiences concerning their approaches to sleep complaints were evaluated through a series of open-ended questions (Table 2), developed to cover the issues of the perceived best practice, the pharmacological and non-pharmacological strategies employed as well as the barriers to delivering what participants perceived as best practice. The questions were designed by a researcher with a wide experience in qualitative research (VG) and by a psychiatrist having worked in that hospital (MDR). Two pilot interviews were conducted in May 2022. Interviews were conducted using a videoconference tool, in the participants’ workplace. Field notes were taken by the researcher during and after the interviews.

### 4.4. Data Analysis

Interviews were analysed using inductive thematic analysis so that the analysis was data-driven [51,52]. Transcribed interviews were manually analysed by MDR, who assigned codes to the content of the interviews, and supervised by VG. Codes were later regrouped into categories that were further regrouped into three overarching themes.

### 4.5. Researcher

The study was conducted by one researcher (MDR), a female junior psychiatrist who had previously worked in the psychiatric hospital of the study, and who was working in the psychiatric outpatient clinic linked to that hospital at the time of the study. This research was part of a Master’s thesis for the obtention of a Master’s in Public Health at King’s College London.

### 4.6. Ethics

The study was reviewed and approved by the King’s College London Ethics Research Committee (Reference: LRU/DP-21/22-26974). All participants gave written informed consent.

## 5. Conclusions

The approaches of psychiatrists and nurses to sleep complaints among inpatients in a Swiss psychiatric hospital were highly heterogeneous and largely not evidence-based, with insufficient adaptation of cognitive behavioral therapy for insomnia (CBT-I) to the inpatient setting. This heterogeneity may be explained by several factors: the broad and diverse nature of “sleep complaints,” the limited applicability of existing insomnia guidelines to hospital settings, their insufficient consideration of the clinical complexity of psychiatric patients, and a general lack of education and training in sleep medicine among psychiatrists and psychiatric nurses. Addressing these factors—and, more importantly, strengthening education and training not only for psychiatrists and psychiatric nurses but also for general physicians and other healthcare professionals—should be considered a priority within the field of sleep medicine.

## Figures and Tables

**Table 1:** Demographic data from the participants.

Participant	Gender	Position	Experience in Psychiatry
P1	Female	Chief nurse	5 to 10 years
P2	Female	Chief psychiatrist	5 to 10 years
P3	Female	Junior psychiatrist	2 to 5 years
P4	Male	Junior psychiatrist	<2 years
P5	Female	Junior psychiatrist	2 to 5 years
P6	Female	Junior psychiatrist	2 to 5 years
P7	Female	Chief psychiatrist	>10 years
P8	Female	Night nurse	>10 years
P9	Male	Chief nurse	>10 years
P10	Female	Night nurse	>10 years

**Table 2:** Open-ended questions for the semi-structured interviews.

1. What would be the best way to act in front of a patient who presented with sleep complaints during a psychiatric hospitalisation?
2. What are the factors that make one act differently between one patient and another?
3. In which situation is one most likely to use a hypnotic medication?
4. What influences the choice of hypnotic medication?
5. In which situation is one most likely to use a non-pharmacological strategy?
6. Which factors complicate the integration of sleep management recommendations?
7. Is there anything you would like to add?

## Data Availability

The interviews of this study are not publicly available due to restrictions imposed by the ethics committee overseeing this research. These restrictions prevent the sharing of any raw or processed data. However, additional information may be made available from the corresponding author on reasonable request and subject to approval by the ethics committee.

## Abbreviations

The following abbreviations are used in this manuscript:

CBT-I	Cognitive-Behavioural Therapy for insomnia
COREQ	Consolidated criteria for reporting qualitative research
